# Episodic Short-Term Recognition Requires Encoding into Visual Working Memory: Evidence from Probe Recognition after Letter Report

**DOI:** 10.3389/fpsyg.2016.01440

**Published:** 2016-09-22

**Authors:** Christian H. Poth, Werner X. Schneider

**Affiliations:** Neuro-Cognitive Psychology, Department of Psychology and Cluster of Excellence Cognitive Interaction Technology, Bielefeld UniversityBielefeld, Germany

**Keywords:** visual working memory, visual attention, episodic memory, object recognition, short-term memory

## Abstract

Human vision is organized in discrete processing episodes (e.g., eye fixations or task-steps). Object information must be transmitted across episodes to enable episodic short-term recognition: recognizing whether a current object has been seen in a previous episode. We ask whether episodic short-term recognition presupposes that objects have been encoded into capacity-limited visual working memory (VWM), which retains visual information for report. Alternatively, it could rely on the activation of visual features or categories that occurs before encoding into VWM. We assessed the dependence of episodic short-term recognition on VWM by a new paradigm combining letter report and probe recognition. Participants viewed displays of 10 letters and reported as many as possible after a retention interval (whole report). Next, participants viewed a probe letter and indicated whether it had been one of the 10 letters (probe recognition). In Experiment 1, probe recognition was more accurate for letters that had been encoded into VWM (reported letters) compared with non-encoded letters (non-reported letters). Interestingly, those letters that participants reported in their whole report had been near to one another within the letter displays. This suggests that the encoding into VWM proceeded in a spatially clustered manner. In Experiment 2, participants reported only one of 10 letters (partial report) and probes either referred to this letter, to letters that had been near to it, or far from it. Probe recognition was more accurate for near than for far letters, although none of these letters had to be reported. These findings indicate that episodic short-term recognition is constrained to a small number of simultaneously presented objects that have been encoded into VWM.

## Introduction

Visual information processing is organized in discrete episodes. This is most evident from the fact that the uptake of visual information is largely limited to eye fixations, discrete periods of stable eye position that are interrupted by fast saccadic eye movements (e.g., Krock and Moore, 2015). However, on a greater time scale, processing episodes can also be defined by steps of sensorimotor actions, other task-demands, and changes in the visual environment (Petersen et al., 2012; Duncan, 2013; Schneider, 2013; Herwig, 2015; Poth et al., 2015; Poth and Schneider, 2016). To remain oriented in time and space and to act guided by vision, visual information from consecutive processing episodes must be linked. This is particularly evident from tasks requiring to recognize that objects (or subjects) have been viewed recently (e.g., Sternberg, 1966; Wickelgren, 1970; Kahana and Sekuler, 2002; Zhou et al., 2004; Donkin and Nosofsky, 2012). For example, imagine you are standing at a busy inner-city intersection and someone shows you a picture of a dog that just went missing and asks if you have seen it. To answer this question, you must be able to recognize if the dog appeared in one of the many recent processing episodes that consisted of your eye fixations, steps of your actions, and periods of cars passing by. Such tasks require *episodic short-term recognition*: the cognitive function of recognizing whether a now-present object has been contained in a recently passed visual processing episode^1^ (cf. Kahana and Sekuler, 2002; Zhou et al., 2004; Donkin and Nosofsky, 2012).

How is episodic short-term recognition accomplished? What are its underlying mechanisms? First of all, to recognize that an object has been present before, the object must be represented internally. Several views on visual processing posit that initially, objects are represented by activating their corresponding feature or category representations in visual long-term memory (Cowan, 1988; Bundesen, 1990; Henderson, 1994; Henderson and Anes, 1994; Eriksson et al., 2015; cf. Oberauer, 2002; LaRocque et al., 2014; for a more general overview, see Palmeri and Tarr, 2008). These representations code for visual features and categories of objects that have been acquired through past visual experience and are often called *visual types* (e.g., Kanwisher, 1987; Kahneman et al., 1992; although other terms are in use as well, e.g., Duncan and Humphreys, 1989; Bundesen, 1990). Visual types represent objects in a multidimensional feature and category space and they may also represent exemplars of certain objects (cf. Kahana and Sekuler, 2002; Nosofsky et al., 2011; Donkin and Nosofsky, 2012).

Critically, activating an object’s visual type (feature, category) is only considered an initial step of processing (Duncan and Humphreys, 1989; Bundesen, 1990; Bundesen et al., 2005; Kyllingsbæk, 2014). This activation does neither suffice to act upon the object nor to consciously perceive the object in the sense that it can be reported. Importantly, the activation is “pre-attentive” in the sense of being unselective: it proceeds likewise for all objects in the visual field (or parts of the visual field, depending on pre-existing spatial biases, Bundesen and Habekost, 2008, p. 117, and retinal inhomogeneity, Strasburger et al., 2011). That is, it proceeds before mechanisms of visual attention select task-relevant objects for further processing at the expense of task-irrelevant ones (e.g., Duncan and Humphreys, 1989; Bundesen, 1990; Bundesen et al., 2005; Duncan, 2006; Poth et al., 2014). For action and report, objects must be attended, processed further, and eventually encoded into visual working memory (VWM; Duncan and Humphreys, 1989; Bundesen, 1990; Cowan, 2001; Bundesen et al., 2005; Schneider, 2013; note that we use VWM synonymously to the also common term of visual short-term memory).

Visual working memory consists of a mechanism for retaining visual object representations accessible over short time-windows (for reviews, see Luck, 2008; Bundesen et al., 2011; Luck and Vogel, 2013; LaRocque et al., 2014; Ma et al., 2014). In this way, VWM may provide an essential basis for further processing these representations, as recoding them into other representational formats (e.g., the verbal format) so that they can be retained and used by non-visual mechanisms of working memory (e.g., Logie, 2011). The capacity of VWM is limited so that it can only hold about three to four objects (e.g., Sperling, 1960; Shibuya and Bundesen, 1988; Luck and Vogel, 1997; Dyrholm et al., 2011; Poth et al., 2014; note that capacity is also limited in the number of object features, Wheeler and Treisman, 2002; Oberauer and Eichenberger, 2013, and the precision of object features, Wilken and Ma, 2004; Bays and Husain, 2008). Which of all available objects are encoded into VWM depends on selection by visual attention (e.g., Duncan and Humphreys, 1989; Bundesen, 1990; Bundesen et al., 2005; Duncan, 2006; Poth et al., 2014). Because of the limited capacity of VWM, all visually available objects may initially and (pre-attentively) activate visual types in visual long-term memory, but only a limited number of objects is (attentively) processed up to the level of VWM (Duncan and Humphreys, 1989; Bundesen, 1990; Bundesen et al., 2005). Encoding objects into VWM is a core requirement of visually controlled behavior, because objects can only be reported and used for action when they are represented in VWM (Duncan and Humphreys, 1989; Bundesen, 1990; Bundesen et al., 2005). This paper focuses on the open question of whether encoding into VWM is also necessary for episodic short-term recognition.

Episodic short-term recognition requires comparisons of object representations of a recently preceding processing episode with representations of objects of the current episode. This can be conceptualized as a decision process (e.g., Pearson et al., 2014) which is driven by the degree of similarity between these two kinds of representations (e.g., Ratcliff, 1978; Donkin and Nosofsky, 2012; cf. Kahana and Sekuler, 2002). Two rival hypotheses can be advanced regarding the role of VWM in this comparison process (based on the literature covered above). According to the *VWM-encoding* hypothesis, episodic short-term recognition of an object from a previous episode requires that the object has been encoded into VWM. Consequently, objects that have not been processed up to the level of VWM cannot be used for episodic short-term recognition. Alternatively, the *type-activation* hypothesis states that episodic short-term recognition is also possible for objects which have not been encoded into VWM but whose mere presentation has activated their visual types in visual long-term memory. This means that episodic short-term recognition is possible for all external objects that have been visually available within recent eye fixations. In such a case, activations of visual types could extend into the next processing episode. These remaining activations could be matched against activations elicited by objects of this episode. A resulting signal could then allow the comparison of object representations from the previous episode and from the actual environment underlying episodic short-term recognition (e.g., Ratcliff, 1978; Donkin and Nosofsky, 2012). Such a mechanism could be similar to mechanisms assumed to produce attention-independent priming effects, where the presentation of objects facilitates their subsequent object recognition (e.g., Kahneman et al., 1992; Henderson, 1994; Henderson and Anes, 1994; Jensen and Lisman, 1998) or affects motor responses to other stimuli (even if the objects are not discriminable, Klotz and Neumann, 1999, and hence not in VWM, Bundesen, 1990).

Here, we aimed at deciding between the two hypotheses. In two experiments, we asked whether episodic short-term recognition of an object requires that this object has previously been encoded into capacity-limited VWM. To approach this question, we introduced a new paradigm combining letter report with probe recognition.

## Experiment 1

In Experiment 1, participants performed a whole report task (e.g., Sperling, 1960; Shibuya and Bundesen, 1988) which was combined with a probe recognition task. They briefly viewed displays of to-be-memorized letters (memory letters) and then, after a retention interval, reported as many letters as they could. The retention interval outlasted early sensory memory (e.g., Sperling, 1960; Phillips, 1974; Irwin and Thomas, 2008) so that letter reports should have required retention in VWM (followed by a recoding into a verbal format on which the actual report was based, e.g., Logie, 2011; Baddeley, 2012). Memory letters were always 10 different ones, exceeding VWM capacity and thus ensuring participants could never report all letters (Sperling, 1960; Shibuya and Bundesen, 1988). After reporting the letters, a single probe letter appeared within the same trial and participants indicated whether or not the probe had been shown as one of the previous memory letters. Importantly, the probe was either one of the memory letters *and* reported (*reported condition*), or one of the memory letters but not reported (*non-reported condition*), or it was a letter not contained in the set of memory letters (*not shown condition*).

Here, episodic short-term recognition was assessed as performance in probe recognition, that is, in indicating whether or not the probe letter had been shown as one of the memory letters. Which memory letters were encoded into VWM was assessed by preceding letter reports. Since VWM is defined by the accessibility of its content (e.g., Bundesen, 1990; Bundesen et al., 2005; Schneider, 2013; but see, Soto et al., 2011), reported letters must have been in VWM by definition. Following a number of theories (e.g., Bundesen, 1990; Bundesen et al., 2005; Martens and Wyble, 2010; Schneider, 2013), we assume that letters which were not reported did not enter VWM. Consequently, the VWM-encoding hypothesis predicts higher probe recognition performance in the reported than in the non-reported and not shown conditions. In contrast, no such performance differences are expected based on the type-activation hypothesis. According to this hypothesis, performance should be equal in the reported and non-reported conditions. More specifically, episodic short-term recognition should be possible for all presented memory letters, irrespective of their encoding into VWM. That is because all presented memory letters should have activated their visual types in visual long-term memory as part of the initial processing of the letters (e.g., Duncan and Humphreys, 1989; Bundesen, 1990; Bundesen et al., 2005; Kyllingsbæk, 2014; see above). Besides testing these hypotheses, Experiment 1 explored whether memory letters in the whole report task were encoded in a spatially clustered manner. That is, whether letters in close spatial proximity were encoded with preference over letters that were farther apart. Such a spatial clustering may reveal attentional selection strategies and this will become important in Experiment 2.

### Method

#### Participants

Fourteen participants were paid to take part in the experiment. They were between 18 and 30 years old (*Mdn* = 20 years), nine were male, five female, 13 were right-handed and one left-handed, and all reported normal or corrected-to-normal visual acuity and color vision. All participants gave written informed consent before performing the experiments that were conducted according with the ethical standards of the German Psychological Association (Deutsche Gesellschaft für Psychologie, DGPs), and were approved by Bielefeld University’s ethics committee. One additional participant was excluded from data analysis because of an experimentation error.

#### Apparatus and Stimuli

The experiment took place in a dimly lit room. Stimuli were presented on a 19″ CRT-screen (Trinitron MultiScan G420, Sony, Park Ridge, NJ, using a graphics card of type Quadro NVS 290, NVIDIA, Santa Clara, CA, USA) with a refresh rate of 85 Hz and a resolution of 1280 × 1024 pixels at physical dimensions of 36 cm × 27 cm. The participant’s head was stabilized by a chin rest positioned 71.8 cm from the screen. Responses were collected using a standard computer keyboard with German layout. Labels indicating “yes” (by the German word “Ja”) and “no” (by the German word “Nein”) were placed above the F1 and F9 keys of the keyboard. The experiment was controlled by the Psychophysics Toolbox 3.0.12 extension (Brainard, 1997; Pelli, 1997; Kleiner et al., 2007) for MATLAB R2013b (The MathWorks, Natick, MA, USA).

A MAVOLUX-digital luminance meter (Gossen, Nuremberg, Germany) was used to measure stimulus luminance. Black letter stimuli (0.32° of visual angle × 0.48°; < 1 cd × m^-2^) from the set [ABDEFGHJKLMNOPRSTVXZ] (this set of letters was chosen to avoid highly confusable letters, as e.g., by Poth et al., 2015) were located equally spaced on an imaginary circle with a radius of 2° around screen center. Fixation cross (0.32° × 0.32°) and response screen text were white (108 cd × m^-2^). The response screen showed the German text “Buchstaben?”, which means “Letters?” in English. Stimuli were shown against a gray background (21 cd × m^-2^).

#### Procedure and Design

**FIGURE 1:**
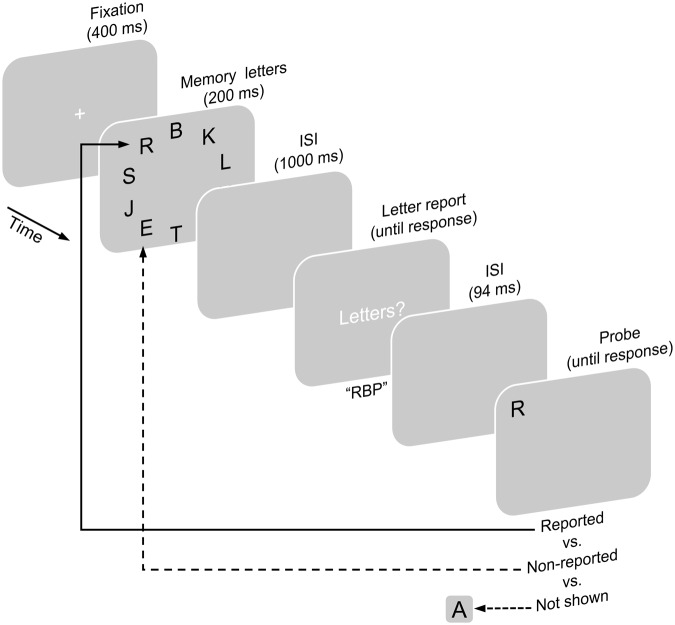
**Experimental paradigm of Experiment 1.** On each trial, participants fixated a fixation cross after which 10 memory letters were shown. After an interstimulus interval (ISI), a response screen appeared (showing the text “Buchstaben?” instead of the here stated translation “Letters?”) and lasted until participants indicated that they finished reporting as many memory letters as they could (whole report task). After another ISI a single probe letter was presented. Participants then indicated whether or not the probe was one of the memory letters (probe recognition task). Probes have been either both, contained in the set of memory letters and reported (reported condition), or contained but not reported (non-reported condition), or they have not been contained (not shown condition).

Before the experiment, participants read instructions on the screen and reported them to the experimenter in their own words. The experimenter repeated the instructions again, if participants had reported them incorrectly. **Figure 1** illustrates the experimental paradigm. Participants initiated each trial by pressing the space-bar. In the beginning of a trial, a fixation cross was shown for 400 ms. Next, 10 memory letters were presented for 200 ms. The letters were randomly drawn without replacement from the set of used letters. The memory letters were followed by a blank interstimulus interval (ISI) lasting for 1000 ms (this duration ensures that early sensory (iconic) memory representations of the letters have been decayed, e.g., Sperling, 1960; Phillips, 1974; Irwin and Thomas, 2008), after which a response screen prompted participants to enter letters. Participants should report as many from the preceding memory letters as they could (without being required to report as many as 10 letters). A maximum of 10 letters could be entered (but this never happened). After confirming that they had finished reporting letters by pressing the enter-key, another ISI of 94 ms followed. Then a single probe letter was presented. Participants indicated whether or not this probe was one of the preceding memory letters by pressing the F1 or F9 key, respectively.

The probe was manipulated in three conditions of a within-subjects design. In the reported condition, the probe was randomly chosen from the letters which were shown and reported by the participant on this trial. In the non-reported condition, the probe was one of the letters that were shown on this trial but that the participant did not report. In both of these two conditions, probes appeared at their locations in the display of the memory letters. In the not shown condition, the probe was randomly chosen from the set of all letters excluding the memory letters of the trial (irrespective of whether participants had entered these letters). In this condition, the probe appeared at a random location.

Participants performed three blocks of 100 trials, each comprising 25 trials of the reported, 25 trials of the non-reported, and 50 trials of the not shown condition. Twice as many trials of the not shown as of the other two conditions were included to equate the number of trials in which a previously shown (correct answer “yes”) or a not shown letter (correct answer “no”) was probed. Within each block, trials of the three conditions were administered in random order. Participants performed twelve training trials prior to the experiment.

### Results and Discussion

A significance criterion of *p* < 0.05 was used for all statistical analyses. Performance in the three conditions was compared using one-way repeated-measures analyses of variance with type II sums-of-squares for which 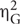 (Bakeman, 2005) is reported as effect size. Where the assumption of sphericity was violated, *p*-values are based on Greenhouse–Geisser-corrected degrees of freedom and the correction factor ε is reported alongside the uncorrected degrees of freedom. Paired *t*-tests (two-tailed) with Bonferroni-corrected *p*-values (*p*_B_) were used for pairwise comparisons for which *d*_z_ (Cohen, 1988) is reported as effect size. These *t-*tests were supplemented with corresponding Bayes factors (*BF*; Rouder et al., 2009), of which values greater one favor the null hypothesis and values smaller one favor the alternative hypothesis. All analyses were performed using R (3.0.3; R Development Core Team, 2016).

A total of 3.3% of all trials were discarded before analysis because either, (1) none of the memory letters was reported (0.57%), or (2) duplicate letters were contained in the letter report (2.76%).

#### Letter Report Performance

Letter report performance was assessed as participants’ mean number of correctly reported letters, that is, for each individual participant the mean number of typed-in letters matching one of the memory letters across trials. There were no significant differences regarding letter report performance in the three conditions, *F*(2,26) = 2.231, *p* = 0.128, 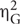 = 0.002. In addition, mean letter report performance was in the range of three to four letters in all three conditions (reported: *M* = 3.62, *SD* = 0.59, min = 2.41, max = 4.60; non-reported: *M* = 3.56, *SD* = 0.61, min = 2.35, max = 4.41; not shown: *M* = 3.56, *SD* = 0.59, min = 2.44, max = 4.5), consistent with previous estimates of VWM capacity in letter report tasks (Sperling, 1960; Shibuya and Bundesen, 1988).

#### Spatial Clustering of Reported Letters

**FIGURE 2:**
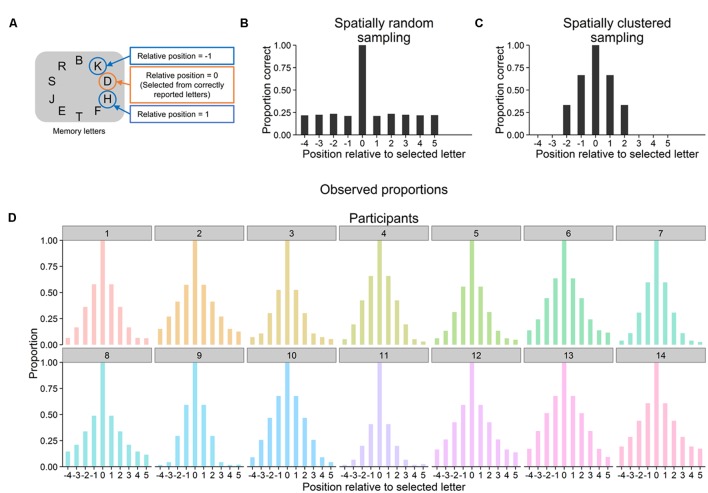
**(A)** For each correctly reported letter, it was determined whether letters at the 10 relative positions in the display of memory letters were reported as well. Averaged across all correctly reported letters, this resulted in a proportion of correctly reported letters for each relative position. **(B)** Simulation of 1000 trials in each of which three letters were randomly sampled from the display of memory letters. Proportions of correctly reported letters are shown as a function of position relative to a selected correctly reported letter (averaged across trials). Proportions are approximately equal for all relative positions except for the position of the selected letter (which must always equal one). **(C)** Simulation of 1000 trials in each of which three letters were sampled that where next to each other in the display of memory letters. Proportions monotonically decrease from relative positions closer to selected letters to relative positions farther away from them. **(D)** For each individual participant, observed proportions of correctly reported letters as a function of position relative to a selected reported letter (across all correctly reported letters and averaged over all trials).

Whether letters were encoded into VWM in a spatially clustered manner was assessed as follows. For each trial, the extent to which reported letters were spatially clustered within the original display of memory letters (i.e., their spatial proximity in this display) was quantified. The data was collapsed across conditions, since trials in the three conditions did not differ until after letters had been reported. Each correctly reported letter was selected for one step of the analysis. For this selected letter, it was determined whether or not the memory letters at the 10 positions relative to it were correctly reported (**Figure 2A**). This must be always the case for relative position zero, as this is the position of the selected letter itself. The procedure resulted in a matrix with the dimensions number of reported letters (rows) × 10 letter positions (columns) and with entries coding for whether or not a given letter has been reported. Now, spatial clustering of letter reports was assessed as the proportions of reported letters for each letter position (i.e., for each column) across all reported letters (i.e., across all rows). If participants reported letters in a spatially random manner, then these proportions should be equal with the exception of a proportion of 1 for the selected letters (see **Figure 2B** for a computer simulation). In contrast, spatial clustering in encoding letters would become manifest in higher proportions for letters at positions more proximal compared with positions more distant to the selected letter (**Figure 2C** for a computer simulation). Note that these analyses require that the number of presented letters clearly exceeds participants’ VWM capacity because otherwise there would be no clear differences between proportions. This condition is assumed to be met because participants reported between three and four of the 10 presented letters (see the letter report performance above).

As can be seen in **Figure 2D**, the mean proportions of reported letters monotonically decreased with increasing distance to selected letters and this pattern was present in all participants. Page’s trend test was used to test whether monotonic decreases from closer to more distant positions were statistically significant. To this end, Page’s trend test was applied to the participants’ proportions at relative positions -1 to -4 and, separately, at relative positions 1 to 5 (**Figure 2A**). Results revealed monotonic decreases for both of these subsets of the data, locations -1 to -4: *L* = 420, *p* < 0.001, locations 1 to 5: *L* = 768, *p* < 0.001 (and these monotonic decreases were present in all of the three blocks of trials, all *L*s > = 420, all *p*s < 0.001).

Selective encoding of letters into VWM was not spatially random. Instead, all participants encoded subsets of the memory letters into VWM that were in close spatial proximity in the letter display. This spatial clustering may reflect an attentional encoding strategy. Participants learned over trials that always more memory letters were shown than they could report. Thus, participants learned they had to select subsets of the memory letters for report. Spatial clustering may be a means to accomplish such a selection from equally task-relevant objects by restricting encoding to objects in close spatial proximity. In this way, spatial clustering may reflect the distribution of spatial attention (e.g., Posner, 1980; Bundesen, 1990), which in this specific case selects objects at or close to a strategically and internally specified location.

#### Probe Recognition Performance

**FIGURE 3:**
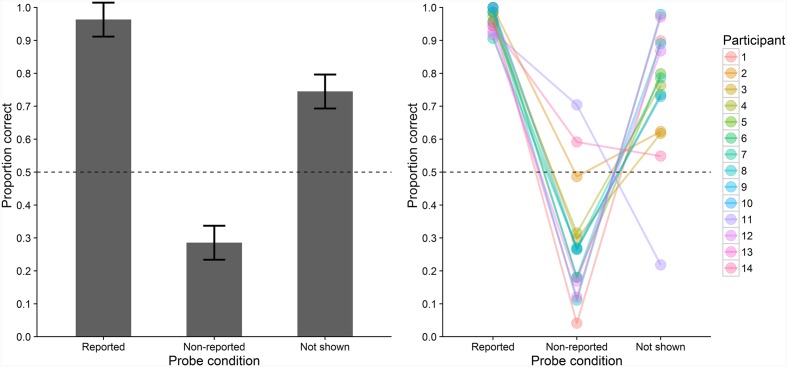
**Mean probe recognition performance (proportions of correct responses to probe letters) in Experiment 1 across all participants (bars, **left**) and for individual participants (colored points, **right**) in the three conditions.** Error-bars indicate ±1 *SE* for within-subjects designs (Loftus and Masson, 1994). Chance level is indicated by the dashed line.

Probe recognition performance was assessed as the proportion of trials on which probe letters were correctly recognized as having been shown or not shown on the trial. **Figure 3** depicts the participants’ probe recognition performance, both at the sample and individual level. Probe recognition performance differed significantly between the three conditions, *F*(2,26) = 44.912, ε = 0.522, *p* < 0.001, 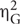 = 0.771. Probe recognition performance was significantly higher in the reported (*M* = 0.96, *SD* = 0.03) compared with the non-reported (*M* = 0.29, *SD* = 0.19), *t*(13) = 12.774, *p*_B_ < 0.001, *d*_z_ = 3.41, *BF* = 8.8 × 10^-7^, and the not shown condition (*M* = 0.74, *SD* = 0.20), *t*(13) = 4.170, *p*_B_ = 0.003, *d*_z_ = 1.11, *BF* = 0.028. Moreover, performance was significantly lower in the non-reported than in the not shown condition, *t*(13) = -4.498, *p*_B_ = 0.002, *d*_z_ = -1.20, *BF* = 0.016. One-sample *t*-tests (two-sided) revealed that performance was significantly below the chance level of 0.5 in the non-reported condition, *t*(13) = -4.243, *p* < 0.001, *BF* = 0.025, whereas it was significantly above chance in the not shown condition, *t*(13) = 4.589, *p* < 0.001, *BF* = 0.014.

Whether probe recognition depended on how many letters participants entered for the whole report (irrespective of whether letters were correct) was assessed as the point-biserial correlation between the number of entered letters and probe recognition performance, separately for each participant and each condition. Values of three participants in the reported condition had to be excluded from this analysis because probe recognition was correct in all trials so that no correlation could be computed. One-sample *t-*tests (two-sided) indicated that the correlations of the 11 remaining participants did not significantly depart from zero in any of the three conditions, all |*t*s| (10) < 1.713, all *p*s > 0.110, all *BF*s > 1.149.

Probe recognition performance was close to ceiling in the reported condition but it was substantially lower in the non-reported and not shown conditions. These findings clearly argue against the type-activation hypothesis which predicts equal performance for all presented memory letters and hence equal performance in the reported and non-reported condition. Instead, the findings seem to support the VWM-encoding hypothesis which predicts higher performance in the reported condition, in which probe letters were encoded into VWM. However, before arriving at these conclusions, several issues should be considered. According to the VWM-encoding hypothesis, performance should have been at chance level in the non-reported condition but it was below chance level. This may indicate that participants based their probe responses not only on the letters they remembered having viewed on this trial. Rather, they may have partly based their responses on the letters they remembered having reported on this trial. This would have biased them away from responding those probes had been contained in the memory letters when they had not reported the letters of these probes. This bias might also have contributed to the above-chance performance in the not shown condition. Besides biasing responses, reporting the letters itself might also have improved their subsequent episodic short-term recognition compared to non-reported letters. Similarly, reporting memory letters might have interfered with retaining non-reported letters. In addition, reporting the letters may have prolonged the interval that the non-reported letters had to be retained. In all of these cases, letters that were inaccessible for report might have been available for later episodic short-term recognition if intervening report requirements were controlled for. Therefore, the aim of Experiment 2 was to control for all effects reporting letters might have on probe recognition performance.

## Experiment 2

**FIGURE 4:**
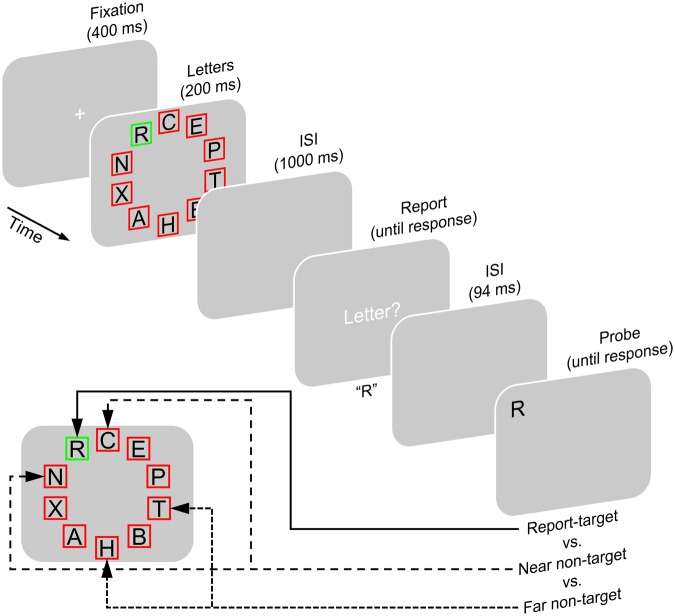
**Experimental paradigm of Experiment 2.** In the beginning of each trial, a fixation cross was shown, after which a display of letters appeared. Each letter was located inside a red or green square. One square differed in its color from the others indicating the report-target, which was to be reported when a response screen appeared after an ISI. Letters in squares of the other color were not to be reported (non-targets). After another ISI, a single probe letter was shown. Then, participants indicated whether or not this probe has been shown as part of the letter display in this trial. The probe either (1) tested the report-target (i.e., was shown at its position and either matched it or had not been presented on this trial; report-target condition), (2) or it tested one of the letters flanking the report-target (near non-target condition), or (3) it tested one of the two letters opposite to the letters flanking the report-target (far non-target condition).

Experiment 2 was designed to investigate episodic short-term recognition performance for letters that were more likely to be encoded into VWM compared with letters whose encoding was less likely. To manipulate the likelihood of encoding specific letters into VWM, we made use of the spatial clustering of VWM encoding found in Experiment 1. Participants briefly viewed a display of 10 letters in which a colored frame identified one letter as report-target and frames in a different color identified the nine other letters as non-targets *regarding report*. Participants’ task was to report the single report-target after a retention interval. After reporting, a single probe letter was shown and participants were to indicate whether or not it had been presented as one of the preceding letters (**Figure 4**). There were three conditions. In the *report-target condition*, the probe tested recognition of the report-target. In the *near non-target condition*, the probe tested recognition of a letter that has been located directly beside the report-target. In the *far non-target condition*, the probe tested recognition of a letter that has been located far away from the report-target, on the other side of the letter display.

The report-target has to be encoded into VWM, in order to be accessible for being reported (e.g., Bundesen, 1990; Bundesen et al., 2005; Schneider, 2013). Because of the spatial clustering of letter reports in Experiment 1, we assumed that while participants aimed at encoding the report-target, they were more likely to encode near non-targets selectively compared with far non-targets. This is compatible with the view that spatial attention was primarily directed at the report-target (e.g., Kim and Cave, 1995; Gaspelin et al., 2015), but was secondarily directed more at near non-targets than at far non-targets or was secondarily directed at near non-targets only. According to the VWM-encoding hypothesis, probe recognition performance should be highest for report-targets, followed by near non-targets, and lowest for far non-targets because of their lowest likelihood of being encoded into VWM. In contrast, according to the type-activation hypothesis probe recognition performance should be equal for all presented letters and thus equal in all three conditions. Importantly, the near and far non-targets were not subject to report requirements.

### Method

#### Participants

Ten paid participants took part in Experiment 2. They were between 22 and 30 years old (*Mdn* = 25). Four of them were male, six female, nine were right, and one left-handed. All participants reported normal or corrected-to-normal visual acuity and color vision. They gave written informed consent before performing the experiments that were conducted according to the ethical standards of the German Psychological Association (DGPs), and were approved by Bielefeld University’s ethics committee.

#### Apparatus and Stimuli

The apparatus and experimental setup of Experiment 2 were the same as those of Experiment 1. The stimuli of Experiment 2 were identical to those of Experiment 1 with the following exceptions. All letters were placed inside a square frame (0.72° × 0.72°). Frames of the nine non-targets were either all red (20 cd × m^-2^; RGB: 255, 0, 0) or green (76 cd × m^-2^; RGB: 0, 255, 0). The frame of the report-target was in the other color (i.e., green when the others were red or red when the others were green). The colors of report-target and non-targets remained the same throughout the experiment. Whether red or green indicated the report-target was counterbalanced across the sample. The text of the response screen was identical to that in Experiment 1, except that it prompted participants to enter only one instead of several letters (by the German text “Buchstabe?”, which means “Letter?” in English).

#### Procedure and Design

As illustrated in **Figure 4**, the experimental paradigm of Experiment 2 was identical to that of Experiment 1 except for the following aspects. Instead of all 10 letters, participants were to report only the one report-target (partial report). On each trial, the position of the report-target was randomly chosen. No confirmation of this report was required, instead the trial proceeded as soon as a letter-key had been pressed. As in Experiment 1, at the end of each trial a single probe letter was shown and participants were required to indicate whether or not it was shown within the letter display of this trial. Participants performed three conditions of a within-subjects design. In the *report-target condition*, the probe appeared at the location of the report-target and either matched the report-target or consisted in a letter not presented on this trial. In the *near non-target condition*, the probe appeared at the location of one of the two letters that flanked the report-target and either matched this letter or had not been presented on this trial. In the *far non-target condition*, the probe appeared at the location of one of the two letters opposite to the two flanking letters, on the other side of the letter display than the report-target and either matched this letter or had not been shown on this trial.

Participants performed four blocks of 72 trials each comprising 24 trials of the report-target, near non-target, and far non-target condition. For the two non-target conditions, probes appeared equally often at positions in clockwise or counter-clockwise direction of the report-target. In each of the three conditions and for each possible probe location, trials with probes matching the former letter at the probe’s location (correct answer “yes”) and probes not shown (correct answer “no”) occurred equally often. Participants performed 24 training trials prior to the experiment.

### Results and Discussion

The same statistical procedures were used as in Experiment 1. Two trials were excluded from analysis because participants entered more than one letter in their letter report (which could happen only if participants pressed two keys close to simultaneously). Whether report-targets were in red or green frames did not interact with any of the below described dependent variables, all *F*s < 1.64, all *p*s > 0.227 (revealed by a repeated-measures ANOVA with type III sums-of-squares). Therefore, data of participants with report-targets in red and green frames was collapsed for the following analyses.

#### Letter Report Performance

Letter report performance was assessed as participants’ proportion of trials on which the report-target was correctly reported. Unsurprisingly, there were no significant differences between letter report performance in the three experimental probe conditions (report-target condition: *M* = 0.94, *SD* = 0.07; near non-target condition: *M* = 0.94, *SD* = 0.06; far non-target condition: *M* = 0.93, *SD* = 0.07), *F*(2,18) = 0.545, *p* = 0.589, 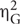 < 0.004. In addition, Friedman’s test was applied, because the assumption of normal distribution of the repeated-measures analysis of variance was not met. This test yielded a non-significant effect as well, χ^2^(2) = 1.316, *p* = 0.518.

Participants’ letter report performance did not differ reliably between the three conditions. Participants achieved close-to-ceiling performance in all three conditions, as could be expected since only one letter had to be reported which should not touch the capacity limit of VWM (Sperling, 1960; Shibuya and Bundesen, 1988).

#### Probe Recognition Performance

Different from Experiment 1, each condition contained trials in which probes did and trials in which probes did not match the letters they referred to. Therefore, probe recognition performance could be quantified as *d*’, the difference between the z-transformed rate of correct responses to probes shown on this trial, *z*(“hit rate”), and the z-transformed rate of false responses to probes not shown on this trial, *z*(“false alarm rate”; for an overview, see Macmillan and Creelman, 2005). Performance at chance level leads to a *d*’ of zero and close to perfect performance to values of 4.65 (or higher and 0.5 was added to all data cells on which hit and false alarm rates were based to avoid infinite values for *d*’, Macmillan and Creelman, 2005, pp. 8–9,). To facilitate comparison with the results of Experiment 1, in **Table 1** we also report the probe recognition performance assessed as the proportion of trials on which probe letters were correctly recognized as having been shown or not shown on the trial.

**Table 1:** Probe recognition performance in the three conditions of Experiment 2 assessed as the proportion of correct responses to the probe.

	*M* (*SD*)	vs. report-target	vs. near non-target
Report-target	0.79 (0.09)	–	–
Near non-target	0.61 (0.08)	*t*(9) = -6.515, *p*_B_ < 0.001, *d*_z_ = -2.06, *BF* = 3.79 × 10^-3^	–
Far non-target	0.51 (0.05)	*t*(9) = -9.313, *p*_B_ < 0.001, *d*_z_ = -2.94, *BF* = 3.30 × 10^-4^	*t*(9) = -3.451, *p*_B_ = 0.022, *d*_z_ = -1.09, *BF* = 0.125


*Provided are means (*M*) with standard deviations (*SD*) in parentheses, and for each of the three conditions (rows), the results of the pairwise comparisons to the other two conditions (last two columns).*

**FIGURE 5:**
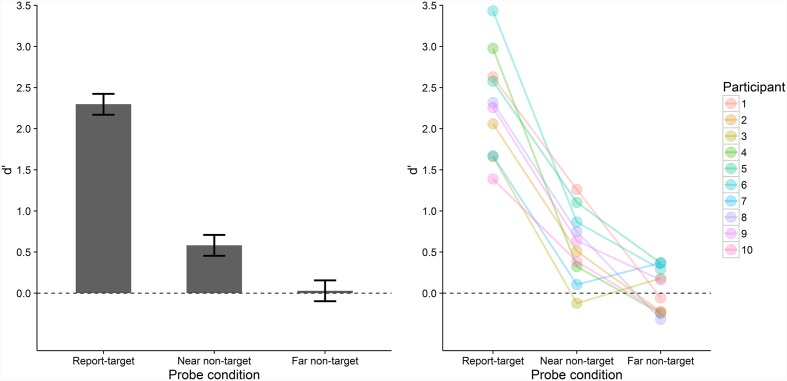
**Mean probe recognition performance (*d*’) in Experiment 2 across all participants (bars, **left**) and for individual participants (colored points, **right**) in the three conditions.** Error-bars indicate ±1 *SE* for within-subjects designs (Loftus and Masson, 1994). Chance level is indicated by the dashed line.

**Figure 5** depicts participants’ probe recognition performance in the three conditions at the sample and individual level. Performance differed significantly between the three conditions, *F*(2, 18) = 86.859, *p* < .001, 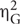 = 0.824. That is, performance was significantly higher in the report-target (*M* = 2.30, *SD* = 0.63) compared with the near non-target (*M* = 0.58, *SD* = 0.43), *t*(9) = 10.562, *p*_B_ < 0.001, *d*_z_ = 3.34, *BF* = 1.3 × 10^-4^, and far non-target condition (*M* = 0.03, *SD* = 0.27), *t*(9) = 10.770 *p*_B_ < 0.001, *d*_z_ = 3.41, *BF* = 1.2 × 10^-4^. Performance was also significantly higher in the near than in the far non-target condition, *t*(9) = 3.435, *p*_B_ = 0.022, *d*_z_ = 1.09, *BF* = 0.127. This data pattern was present in all except two participants whose performance was slightly higher in the far compared with the near non-target condition (**Figure 5**, right). In addition, two one-sample *t*-tests revealed that performance was significantly above chance in the near non-target condition, *t*(9) = 4.262, *p* = 0.002, *BF* = 0.045, but did not differ from chance level (i.e., a *d’* of zero) in the far non-target condition, *t*(9) = 0.335, *p* > 0.745, *BF* = 3.086.

Probe recognition performance was highest when the probe letter tested the former report-target which had been encoded into VWM, as evident from the near-ceiling performance in reporting its identity. Importantly, performance was higher for near non-targets than for far non-targets. This indicates that episodic short-term recognition was better for letters that were more likely to be encoded into VWM compared with letters less likely to be encoded (given that encoding into VWM seems to proceed in a spatially clustered manner, see Experiment 1). In fact, performance for far non-targets was at chance level which suggests that episodic short-term recognition was not possible for these letters. Furthermore, Experiment 2 controlled for potential alternative explanations of the findings of Experiment 1. These alternative explanations stated that differences between the conditions did not stem from whether letters were encoded into VWM but from whether letters were reported. In Experiment 2, near and far non-targets both did not have to be reported and differed only in their distance from the report-target. Hence, the performance difference between these two conditions cannot be attributed to effects reporting letters itself might have on performance. Therefore, we interpret the higher performance for near non-targets compared with the performance at chance level for far non-targets as strong evidence for the VWM-encoding hypothesis. Conversely, we interpret this finding as evidence against the type-activation hypothesis.

## General Discussion

We investigated whether episodic short-term recognition of objects from a previous processing episode requires that these objects have been encoded into VWM. For this purpose, we introduced a new paradigm combining letter report with probe recognition. In two experiments, episodic short-term recognition was assessed as performance in recognizing whether a probe letter was presented in the preceding letter display of the current trial. In Experiment 1, probe recognition performance was higher for letters that had been encoded into VWM compared with letters that had not been encoded. In Experiment 2, only a single letter had to be reported in the letter report task. This controlled for effects reporting letters itself might have on probe recognition. In Experiment 2, probe recognition performance was higher for non-target letters that were near to a report-target letter, and hence more likely to be encoded into VWM, compared with non-target letters far from the report-target, whose encoding was less likely. Crucially, this difference in probe recognition refers to non-target letters which did not have to be reported. Strikingly, performance was at chance level for letters far from the report-target which were unlikely to enter VWM. Therefore, we interpret the present findings as strong evidence for the VWM-encoding hypothesis which states that episodic short-term recognition presupposes that visual objects have been encoded into VWM. Conversely, we interpret these findings as evidence against the type-activation hypothesis. Note that one might distinguish a strong form of the type-activation hypothesis, the one that we have put forward so far, and a weaker form. The strong form states that episodic short-term recognition can be accomplished perfectly (at least in principle) for all objects of the current visual field. In contrast, the weaker form states that episodic short-term recognition can be accomplished for all objects of the visual field, but not perfectly, and that recognition performance may be improved by additional encoding into VWM. The results of Experiment 2 provide evidence against both forms of the type-activation hypothesis. The finding that probe recognition performance was higher for near than for far non-targets argues against the strong form. The finding that performance was at chance level in the far non-target condition argues against the weak form. That is, episodic short-term recognition seemed impossible in this condition. Thus, taken together, the present findings indicate that type-activation is not sufficient for later episodic short-term recognition but that encoding into VWM is required instead.

### Visual Working Memory as a Basis of Episodic Short-Term Recognition

Encoding an object into VWM seems to be necessary for its later episodic short-term recognition. This means that the functional basis of episodic short-term recognition emerges at a level of processing after the activation of visual types in visual long-term memory (e.g., Kanwisher, 1987; Kahneman et al., 1992; cf. Schneider, 1995) and after visual attention has mediated selective encoding into VWM (Duncan and Humphreys, 1989; Bundesen, 1990; Bundesen et al., 2005; Schneider, 2013). In the present study, letters were used as visual objects. After successful visually based recognition, letters can be processed verbally, which makes it likely that their episodic short-term recognition also involved verbal processing in addition to visual processing. However, because the letters had to be acquired visually, they had to be encoded into VWM first, before such a verbal processing could take place. After their encoding into VWM, they may have been recoded into a verbal format. Such a verbal format may have provided the advantage of verbal rehearsal by verbal working memory, which may have prolonged and secured their retention (e.g., Logie, 2011; Baddeley, 2012). Thus, importantly, even though episodic short-term recognition may rely on several different (working) memory mechanisms (such as visual and verbal ones), encoding into VWM seems to be a necessary processing step for these mechanisms to operate.

Why may encoding into VWM be necessary for episodic short-term recognition? Several theories assume that by encoding into VWM, information about visual objects is transformed into a special representational state (e.g., Cowan, 1988; Oberauer, 2002; LaRocque et al., 2014; cf. Olivers et al., 2011). We suggest that it is this representational state that makes encoding into VWM a requirement of episodic short-term recognition. Specifically, we propose that two characteristics of this representational state are necessary for episodic short-term recognition: *binding* and *robustness*.

Binding means that different visual features of an object are integrated which yields representations of objects as a whole, with all their features (e.g., Treisman and Gelade, 1980). The mere presentation of objects activates visual types (features) in visual long-term memory but this happens in isolation (cf. Bundesen, 1990; Schneider, 1995). Episodic short-term recognition requires binding of activated visual types because otherwise objects that share visual features cannot be distinguished. VWM is assumed to mark the first level in the course of visual processing at which the visual types (or features) activated by an object are bound to integrated object representations (Bundesen, 1990; Luck, 2008; Schneider, 2013; Kyllingsbæk, 2014). This point is illustrated by referring to integrated object representations as VWM objects (Schneider, 2013), which have also been called object files (Kahneman et al., 1992) and visual tokens (Schneider, 1995). In sum, the binding of visual types within object representations in VWM may be one reason for that episodic short-term recognition requires encoding into VWM.

Robustness means that object representations in VWM are protected against so-called proactive interference (Keppel and Underwood, 1962). Proactive interference arises when the same visual objects occur repeatedly (e.g., Endress and Potter, 2014). It describes an impairment in recognizing if an object has been viewed in the very recent past as opposed to having been encountered before at all (e.g., Endress and Potter, 2014). Episodic short-term recognition clearly requires to assess whether an object has been viewed in a recently passed episode rather than at some unspecified point in the past. Hence, successful episodic short-term recognition presupposes that proactive interference is eliminated. Robustness against proactive interference is assumed to be a hallmark of VWM representations and providing it is considered a core function of VWM (Endress and Potter, 2014). Thus, taken together, episodic short-term recognition may presuppose encoding of objects into VWM because this might establish representations of objects as bound units (cf. Luck and Vogel, 1997) which are robust against proactive interference (cf. Endress and Potter, 2014).

### Episodic Short-Term Recognition Might Be Constrained by an Encoding-Limitation but Not a Retention-Limitation of Visual Working Memory

As we have argued, the present findings indicate that episodic short-term recognition presupposes encoding into VWM but this seems to conflict with earlier findings. Specifically, Sternberg (1966) presented participants with series of up to six digits followed by a probe digit. Participants indicated whether the probe was contained in a given series. The six presented digits exceed the number of about three to four objects that VWM can hold (e.g., Sperling, 1960; Shibuya and Bundesen, 1988; Luck and Vogel, 1997). Thus, when the last two digits were shown, VWM should have already been filled up so that the digits could not be encoded into VWM. Nevertheless, Sternberg found that probe recognition performance was close to ceiling even for six digits. One might attribute this result to the relatively long presentation durations of digits (1.2 s) that could have allowed verbal rehearsal (e.g., Sternberg, 1975). However, congruent to Sternberg’s findings, later experiments revealed high levels of probe recognition performance for objects that were presented more briefly and thus difficult to rehearse verbally (Endress and Potter, 2014). Taken together, these findings are compatible with the type-activation hypothesis in that they suggest episodic short-term recognition is possible also for objects that have not reached VWM.

How may the conflict between the present and Sternberg’s (1966; cf. Endress and Potter, 2014) findings be resolved? One solution is provided by Schneider’s (2013) recent “theory of task-driven visual attention and working memory” (TRAM) which offers an account of how visual information processing might be accomplished within and across processing episodes. According to TRAM, a new processing episode is started with each onset of visual objects (e.g., after a saccadic eye movement). A processing episode comprises three phases. Premising upon Bundesen’s (1990) theory of visual attention (a model of biased competition, Desimone and Duncan, 1995), TRAM’s first two phases describe how visual attention mediates selective encoding of visual objects into capacity-limited VWM. In TRAM’s third phase, objects that have been encoded into initial activation-based VWM (i.e., VWM based on persistent neural activity) are consolidated which results in *passive* VWM representations (which do not require neural activity but may rely on short-term changes in synaptic connectivity, as reviewed by Eriksson et al., 2015; Postle, 2015; and Stokes, 2015). Critically, according to TRAM, the number of passive VWM representations is not constrained by the traditionally assumed capacity-limitation of VWM. With this in mind, one may interpret classical estimates of VWM capacity (Sperling, 1960; Shibuya and Bundesen, 1988; Luck and Vogel, 1997) as reflecting an encoding limitation but not a retention limitation. In other words, classical VWM capacity may constrain the amount of object information that can be acquired within one processing episode but not the amount of information that can be retained across episodes. In Sternberg’s (1966) paradigm, each of the serially presented digits should have started a new processing episode. Within each of these episodes, a passive VWM representation of the digit should have emerged. Probe recognition should then have been based on a comparison of these passive VWM representations with actual probe digits (which could involve retrieving passive representations again into classical activation-based VWM; Schneider, 2013). In this vein, episodic short-term recognition becomes possible for more serially presented objects than classical VWM can retain. In contrast, TRAM posits that if several objects are presented simultaneously, as in the present experiments, then this can reach the encoding limit of VWM. All simultaneously presented objects are processed within the same processing episode. Therefore, encoding further objects becomes impossible if activation-based VWM is filled up. Critically, creating passive VWM representations of objects presupposes that the objects have been encoded into VWM. Thus, in a given processing episode, only as many objects as VWM can hold can be consolidated into passive VWM representations. As a consequence, episodic short-term recognition across successive processing episodes should be limited with respect to the number of simultaneously shown objects that can be encoded into VWM. In contrast, episodic short-term recognition should not be restricted with respect to the number of retained objects in VWM because this includes also passive VWM representations that have arisen over the course of several episodes, as in Sternberg’s experiments. Interestingly, recent findings might suggest that in such situations of serial object presentations (RSVP), the capacity of passive VWM can be extended beyond “magical number four” by eliminating proactive interference (Endress and Potter, 2014). As an alternative to consolidation in passive VWM, representations of objects in classical VWM could also be recoded into a different representational format (Petersen et al., 2012) which might then be used for later episodic short-term recognition. The objects of the present experiments consisted of letters which may have been recoded into the verbal format (that is open to verbal rehearsal, e.g., Sternberg, 1975, and may allow retention by working memory systems dedicated to verbal information, e.g., Baddeley, 2012). However, since the to-be-recoded object information is acquired visually, recoding would still presuppose encoding into VWM (Petersen et al., 2012). Hence, episodic short-term recognition would still be constrained by the encoding limitation of VWM but not by a retention limit. However, testing this hypothesis is left for further experimental studies.

## Conclusion

The present study shows that episodic short-term recognition of objects from previous episodes presupposes that the objects have been processed up to the level of VWM. In this way, VWM not only provides bound visual objects for online perception and action within a processing episode but also paves the way for episodic short-term recognition across episodes. However, this also implies that episodic short-term recognition is only possible for a limited number of simultaneously presented objects due to the encoding limitation of VWM (Schneider, 2013; cf. Sperling, 1960; Shibuya and Bundesen, 1988; Luck and Vogel, 1997).

## Author Contributions

CP and WS designed the research. CP programmed the experiments and analyzed the data. CP and WS wrote the paper.

## Conflict of Interest Statement

The authors declare that the research was conducted in the absence of any commercial or financial relationships that could be construed as a potential conflict of interest.

## Footnotes

1 Note that the term episodic short-term recognition refers to the described cognitive function (in the sense of a cognitive task-requirement). In this way, the concept of episodic short-term recognition does not include any assumptions about the cognitive mechanisms enabling to fulfill this function (such as for example, interacting mechanisms of episodic long-term memory or working memory).

## References

[B1] Baddeley A. (2012). Working memory: theories, models, and controversies. *Annu. Rev. Psychol.* 63 1–29. 10.1146/annurev-psych-120710-100422 21961947

[B2] Bakeman R. (2005). Recommended effect size statistics for repeated measures designs. *Behav. Res. Methods* 37 379–384. 10.3758/BF03192707 16405133

[B3] Bays P. M. Husain M. (2008). Dynamic shifts of limited working memory resources in human vision. *Science* 321 851–854. 10.1126/science.1158023 18687968 PMC2532743

[B4] Brainard D. H. (1997). The psychophysics toolbox. *Spat. Vis.* 10 433–436. 10.1163/156856897X00357 9176952

[B5] Bundesen C. (1990). A theory of visual attention. *Psychol. Rev.* 97 523–547. 10.1037/0033-295X.97.4.523 2247540

[B6] Bundesen C. Habekost T. (2008). *Principles of Visual Attention: Linking Mind and Brain*. Oxford: Oxford University Press.

[B7] Bundesen C. Habekost T. Kyllingsbæk S. (2005). A neural theory of visual attention: bridging cognition and neurophysiology. *Psychol. Rev.* 112 291–328. 10.1037/0033-295X.112.2.291 15783288

[B8] Bundesen C. Habekost T. Kyllingsbæk S. (2011). A neural theory of visual attention and short-term memory (NTVA). *Neuropsychologia* 49 1446–1457. 10.1016/j.neuropsychologia.2010.12.006 21146554

[B9] Cohen J. (1988). *Statistical Power Analysis for the Behavioral Sciences*, 2nd Edn New York, NY: Psychology Press.

[B10] Cowan N. (1988). Evolving conceptions of memory storage, selective attention, and their mutual constraints within the human information-processing system. *Psychol. Bull.* 104 163–191. 10.1037/0033-2909.104.2.163 3054993

[B11] Cowan N. (2001). The magical number 4 in short-term memory: a reconsideration of mental storage capacity. *Behav. Brain Sci.* 24 87–185. 10.1017/S0140525X01003922 11515286

[B12] Desimone R. Duncan J. (1995). Neural mechanisms of selective visual attention. *Annu. Rev. Neurosci.* 18 193–222. 10.1146/annurev.ne.18.030195.001205 7605061

[B13] Donkin C. Nosofsky R. M. (2012). The structure of short-term memory scanning: an investigation using response time distribution models. *Psychon. Bull. Rev.* 19 363–394. 10.3758/s13423-012-0236-8 22441957

[B14] Duncan J. (2006). EPS Mid-Career Award 2004: brain mechanisms of attention. *Q. J. Exp. Psychol.* 59 2–27. 10.1080/17470210500260674 16556554

[B15] Duncan J. (2013). The structure of cognition: attentional episodes in mind and brain. *Neuron* 80 35–50. 10.1016/j.neuron.2013.09.015 24094101 PMC3791406

[B16] Duncan J. Humphreys G. W. (1989). Visual search and stimulus similarity. *Psychol. Rev.* 96 433–458. 10.1037/0033-295X.96.3.433 2756067

[B17] Dyrholm M. Kyllingsbæk S. Espeseth T. Bundesen C. (2011). Generalizing parametric models by introducing trial-by-trial parameter variability: the case of TVA. *J. Math. Psychol.* 55 416–429. 10.1016/j.jmp.2011.08.005

[B18] Endress A. D. Potter M. C. (2014). Large capacity temporary visual memory. *J. Exp. Psychol. Gen.* 143 548–565. 10.1037/a0033934 23937181 PMC3974584

[B19] Eriksson J. Vogel E. K. Lansner A. Bergström F. Nyberg L. (2015). Neurocognitive architecture of working memory. *Neuron* 88 33–45. 10.1016/j.neuron.2015.09.020 26447571 PMC4605545

[B20] Gaspelin N. Leonard C. J. Luck S. J. (2015). Direct evidence for active suppression of salient-but-irrelevant sensory inputs. *Psychol. Sci.* 26 1740–1750. 10.1177/0956797615597913 26420441 PMC4922750

[B21] Henderson J. M. (1994). Two representational systems in dynamic visual identification. *J. Exp. Psychol. Gen.* 123 410–426. 10.1037/0096-3445.123.4.410 7996123

[B22] Henderson J. M. Anes M. D. (1994). Roles of object-file review and type priming in visual identification within and across eye fixations. *J. Exp. Psychol. Hum. Percept. Perform.* 20 826–839. 10.1037/0096-1523.20.4.826 8083637

[B23] Herwig A. (2015). Linking perception and action by structure or process? Toward an integrative perspective. *Neurosci. Biobehav. Rev.* 52 105–116. 10.1016/j.neubiorev.2015.02.013 25732773

[B24] Irwin D. E. Thomas L. E. (2008). “Visual sensory memory,” in *Visual Memory*, eds Luck S. J. Hollingworth A. (Oxford: Oxford University Press), 9–41.

[B25] Jensen O. Lisman J. E. (1998). An oscillatory short-term memory buffer model can account for data on the Sternberg task. *J. Neurosci.* 18 10688–10699. 9852604 10.1523/JNEUROSCI.18-24-10688.1998 PMC6793327

[B26] Kahana M. J. Sekuler R. (2002). Recognizing spatial patterns: a noisy exemplar approach. *Vision Res.* 42 2177–2192. 10.1016/S0042-6989(02)00118-9 12207978

[B27] Kahneman D. Treisman A. Gibbs B. J. (1992). The reviewing of object files: object-specific integration of information. *Cogn. Psychol.* 24 175–219. 10.1016/0010-0285(92)90007-O 1582172

[B28] Kanwisher N. G. (1987). Repetition blindness: type recognition without token individuation. *Cognition* 27 117–143. 10.1016/0010-0277(87)90016-3 3691023

[B29] Keppel G. Underwood B. J. (1962). Proactive inhibition in short-term retention of single items. *J. Verbal Learning Verbal Behav.* 1 153–161. 10.1016/S0022-5371(62)80023-1

[B30] Kim M.-S. Cave K. R. (1995). Spatial attention in visual search for features and feature conjunctions. *Psychol. Sci.* 6 376–380. 10.1111/j.1467-9280.1995.tb00529.x

[B31] Kleiner M. Brainard D. Pelli D. (2007). What’s new in Psychtoolbox-3? *Perception* 36 1–16.

[B32] Klotz W. Neumann O. (1999). Motor activation without conscious discrimination in metacontrast masking. *J. Exp. Psychol. Hum. Percept. Perform.* 25 976–992. 10.1037/0096-1523.25.4.976

[B33] Krock R. M. Moore T. (2015). The influence of gaze control on visual perception: eye movements and visual stability. *Cold Spring Harb. Symp. Quant. Biol.* 79 123–130. 10.1101/sqb.2014.79.024836 25752313

[B34] Kyllingsbæk S. (2014). *Modeling Visual Attention: Encoding, Attention, and Short-Term Memory*. Doctoral dissertation, University of Copenhagen, Copenhagen.

[B35] LaRocque J. J. Lewis-Peacock J. A. Postle B. R. (2014). Multiple neural states of representation in short-term memory? It’s a matter of attention. *Front. Hum. Neurosci.* 8:5 10.3389/fnhum.2014.00005 PMC3899521 24478671

[B36] Loftus G. R. Masson M. E. (1994). Using confidence intervals in within-subject designs. *Psychon. Bull. Rev.* 1 476–490. 10.3758/BF03210951 24203555

[B37] Logie R. H. (2011). The functional organization and capacity limits of working memory. *Curr. Dir. Psychol. Sci.* 20 240–245. 10.1177/0963721411415340

[B38] Luck S. J. (2008). “Visual short-term memory,” in *Visual Memory*, eds Luck S. J. Hollingworth A. (Oxford: Oxford University Press), 43–83.

[B39] Luck S. J. Vogel E. K. (1997). The capacity of visual working memory for features and conjunctions. *Nature* 390 279–281. 10.1038/36846 9384378

[B40] Luck S. J. Vogel E. K. (2013). Visual working memory capacity: from psychophysics and neurobiology to individual differences. *Trends Cogn. Sci.* 17 391–400. 10.1016/j.tics.2013.06.006 23850263 PMC3729738

[B41] Ma W. J. Husain M. Bays P. M. (2014). Changing concepts of working memory. *Nat. Neurosci.* 17 347–356. 10.1038/nn.3655 24569831 PMC4159388

[B42] Macmillan N. A. Creelman C. D. (2005). *Detection Theory: A User’s Guide*. New York, NY: Psychology Press.

[B43] Martens S. Wyble B. (2010). The attentional blink: past, present, and future of a blind spot in perceptual awareness. *Neurosci. Biobehav. Rev.* 34 947–957. 10.1016/j.neubiorev.2009.12.005 20025902 PMC2848898

[B44] Nosofsky R. M. Little D. R. Donkin C. Fific M. (2011). Short-term memory scanning viewed as exemplar-based categorization. *Psychol. Rev.* 118 280–315. 10.1037/a0022494 21355662 PMC3136045

[B45] Oberauer K. (2002). Access to information in working memory: exploring the focus of attention. *J. Exp. Psychol. Learn. Mem. Cogn.* 28 411–421. 10.1037//0278-7393.28.3.411 12018494

[B46] Oberauer K. Eichenberger S. (2013). Visual working memory declines when more features must be remembered for each object. *Mem. Cogn.* 41 1212–1227. 10.3758/s13421-013-0333-6 23716004

[B47] Olivers C. N. L. Peters J. Houtkamp R. Roelfsema P. R. (2011). Different states in visual working memory: when it guides attention and when it does not. *Trends Cogn. Sci.* 15 327–334. 10.1016/j.tics.2011.05.004 21665518

[B48] Palmeri T. J. Tarr M. J. (2008). “Visual object perception and long-term memory,” in *Visual Memory*, eds Luck S. J. Hollingworth A. (Oxford: Oxford University Press), 163–207.

[B49] Pearson B. Raskevicius J. Bays P. M. Pertzov Y. Husain M. (2014). Working memory retrieval as a decision process. *J. Vis.* 14 2 10.1167/14.2.2 PMC3912875 24492597

[B50] Pelli D. G. (1997). The VideoToolbox software for visual psychophysics: transforming numbers into movies. *Spat. Vis.* 10 437–442. 10.1163/156856897X00366 9176953

[B51] Petersen A. Kyllingsbæk S. Bundesen C. (2012). Measuring and modeling attentional dwell time. *Psychon. Bull. Rev.* 19 1029–1046. 10.3758/s13423-012-0286-y 22847596

[B52] Phillips W. A. (1974). On the distinction between sensory storage and short-term visual memory. *Percept. Psychophys.* 16 283–290. 10.3758/BF03203943

[B53] Posner M. I. (1980). Orienting of attention. *Q. J. Exp. Psychol.* 32 3–25. 10.1080/00335558008248231 7367577

[B54] Postle B. R. (2015). The cognitive neuroscience of visual short-term memory. *Curr. Opin. Behav. Sci.* 1 40–46. 10.1016/j.cobeha.2014.08.004 26516631 PMC4621097

[B55] Poth C. H. Herwig A. Schneider W. X. (2015). Breaking object correspondence across saccadic eye movements deteriorates object recognition. *Front. Syst. Neurosci.* 9:176 10.3389/fnsys.2015.00176 PMC4685059 26732235

[B56] Poth C. H. Petersen A. Bundesen C. Schneider W. X. (2014). Effects of monitoring for visual events on distinct components of attention. *Front. Psychol.* 5:930 10.3389/fpsyg.2014.00930 PMC4140074 25191303

[B57] Poth C. H. Schneider W. X. (2016). Breaking object correspondence across saccades impairs object recognition: the role of color and luminance. *J. Vis.* 16 1–12. 10.1167/16.11.1 28558391

[B58] R Development Core Team (2016). *R: A Language and Environment for Statistical Computing*. Vienna: R foundation for statistical computing.

[B59] Ratcliff R. (1978). A theory of memory retrieval. *Psychol. Rev.* 85 59–108. 10.1037/0033-295X.85.2.59

[B60] Rouder J. N. Speckman P. L. Sun D. Morey R. D. Iverson G. (2009). Bayesian t tests for accepting and rejecting the null hypothesis. *Psychon. Bull. Rev.* 16 225–237. 10.3758/PBR.16.2.225 19293088

[B61] Schneider W. X. (1995). VAM: a neuro-cognitive model for visual attention control of segmentation, object recognition, and space-based motor action. *Vis. Cogn.* 2 331–376. 10.1080/13506289508401737

[B62] Schneider W. X. (2013). Selective visual processing across competition episodes: a theory of task-driven visual attention and working memory. *Philos. Trans. R. Soc. Lond. B Biol. Sci.* 368:20130060 10.1098/rstb.2013.0060 PMC3758203 24018722

[B63] Shibuya H. Bundesen C. (1988). Visual selection from multielement displays: measuring and modeling effects of exposure duration. *J. Exp. Psychol. Hum. Percept. Perform.* 14 591–600. 10.1037/0096-1523.14.4.591 2974870

[B64] Soto D. Mäntylä T. Silvanto J. (2011). Working memory without consciousness. *Curr. Biol.* 21 R912–R913. 10.1016/j.cub.2011.09.049 22115455

[B65] Sperling G. (1960). The information available in brief visual presentations. *Psychol. Monogr.* 74 1–29. 10.1037/h0093759

[B66] Sternberg S. (1966). High-speed scanning in human memory. *Science* 153 652–654. 10.1126/science.153.3736.652 5939936

[B67] Sternberg S. (1975). Memory scanning: new Findings and current controversies. *Q. J. Exp. Psychol.* 27 1–32. 10.1080/14640747508400459

[B68] Stokes M. G. (2015). ‘Activity-silent’ working memory in prefrontal cortex: a dynamic coding framework. *Trends Cogn. Sci.* 19 394–405. 10.1016/j.tics.2015.05.004 26051384 PMC4509720

[B69] Strasburger H. Rentschler I. Jüttner M. (2011). Peripheral vision and pattern recognition: a review. *J. Vis.* 11 13 10.1167/11.5.13 PMC11073400 22207654

[B70] Treisman A. Gelade G. (1980). A feature-integration theory of attention. *Cogn. Psychol.* 12 97–136. 10.1016/0010-0285(80)90005-5 7351125

[B71] Wheeler M. E. Treisman A. M. (2002). Binding in short-term visual memory. *J. Exp. Psychol. Gen.* 131 48–64. 10.1037/0096-3445.131.1.48 11900102

[B72] Wickelgren W. A. (1970). Time, interference, and rate of presentation in short-term recognition memory for items. *J. Math. Psychol.* 7 219–235. 10.1016/0022-2496(70)90045-3

[B73] Wilken P. Ma W. J. (2004). A detection theory account of change detection. *J. Vis.* 4 1120–1135. 10.1167/4.12.11 15669916

[B74] Zhou F. Kahana M. J. Sekuler R. (2004). Short-term episodic memory for visual textures: a roving probe gathers some memory. *Psychol. Sci.* 15 112–118. 10.1111/j.0963-7214.2004.01502007.x 14738518

